# Extracting the Heterogeneous 3D Structure of Molecular Films Using Higher Dimensional SFG Microscopy

**DOI:** 10.1021/acs.jpclett.4c02679

**Published:** 2024-10-22

**Authors:** Alexander P. Fellows, Ben John, Martin Wolf, Martin Thämer

**Affiliations:** Fritz-Haber-Institut der Max-Planck-Gesellschaft, Faradayweg 4-6, Berlin 14195, Germany

## Abstract

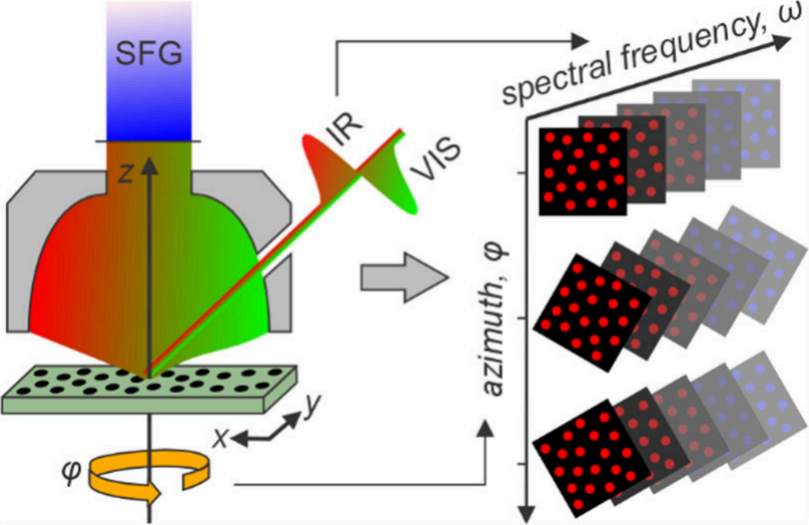

Ultrathin molecular films are widespread in both natural and industrial settings, where details of the molecular structure such as density, out-of-plane tilt angles, and in-plane directionality determine their physicochemical properties. Many of these films possess important molecular-to-macroscopic heterogeneity in these structural parameters, which have traditionally been difficult to characterize. Here, we show how extending sum-frequency generation (SFG) microscopy measurements to higher dimensionality by azimuthal-scanning can extract the spatial variation in the three-dimensional molecular structure at an interface. We extend the commonly applied theoretical assumptions used to analyze SFG signals to the study of systems possessing in-plane anisotropy. This theoretical framework is then applied to a phase-separated mixed lipid monolayer to investigate the variation in molecular density and 3D orientation across the chirally packed lipid domains. The results show little variation in out-of-plane structure but a distinct micron-scale region at the domain boundaries with a reduction in both density and in-plane ordering.

Thin molecular films play an important role in many processes across systems ranging from atmospheric aerosols, the oceanic surface, and physiological membranes, to anticorrosion or tribological coatings and even lab-on-a-chip devices.^1−4^ Therefore, not only are such films highly abundant, but they also have relevance in both natural and industrial settings. The specific impact that these molecular layers have in these systems is critically dependent on their structure, from the macroscopic down to the molecular level, where variations in properties such as molecular density, conformational packing order, and specific composition of different constituents crucially govern their macroscopic behavior and function. In order to understand the mechanisms underlying the processes in these important and omnipresent systems, it is hence necessary to elucidate the structure of these molecular coatings.

One common approach to access many of these critical structural details in molecular films is through vibrational sum-frequency generation (SFG) spectroscopy.^5−18^ The characteristic resonant features in SFG from different functional groups has relevance e.g. for molecular recognition, while subtle changes in the specific line-shape of these features (e.g., center frequency and bandwidth) can be indicative of environmental shifts.^19,20^ Furthermore, polarization-dependent measurements can then also yield orientational information such as molecular tilt angles.^21,22^ One of the most important aspects of SFG, however, is associated with its second-order origin, namely that the sign of the output field is dependent on the absolute molecular orientation (under the electric dipole approximation).^23,24^ This unique symmetry selection rule thus also allows for determination of the absolute molecular directionality within the film (e.g., pointing “up’/‘down” and pointing “left”/“right”) as well as information on the conformational structure of the molecules, e.g., any deviations from “all-trans” chains in alkyl tail groups.^22^

A common feature of typical spectroscopic measurements, however, is their spatial averaging, meaning any kind of structural information associated with micron-scale heterogeneity in the film is lost. While polarization-dependent SFG measurements can yield information on the width of the orientational distribution of particular moieties,^25^ this still lacks insight into the spatial variation across a sample. Given that the vast majority of both natural and synthetic films are heterogeneous at the molecular-to micron-scale, spectroscopic measurements are missing out on a significant part of the structural information that can be critical in determining the overall properties and functionality. Clear examples of this are chemical reaction processes that can be dominated at specific structural sites or domain boundaries,^26^ or cell membranes which contain phase-separated regions named lipid rafts with different molecular composition, packing structure, and density, that at believed to be at the heart of many signaling pathways.^27,28^

The abundance of such heterogeneity hence calls for microscopic SFG imaging of the molecular structure. While spectroscopic imaging tools do not completely remove spatial averaging, they can, in principle, be performed down to the diffraction limit. In consequence, spatial averaging can be reduced to a diffraction-limited spot size, and thus not only uncover general structural heterogeneity at the micro-to-nanoscale, but also reveal information on local molecular directionalities. In this context, vibrational SFG imaging particularly benefits (cf. IR imaging) as the vibrational information is obtained through the detection of visible wavelengths, thus making it possible to achieve submicron resolution. Overall, therefore, vibrational SFG imaging represents an enticing method for structural characterization of thin films as the comprehensive combination of structural properties that are generally accessible through spectroscopic measurements can be further combined with any information on their microscale heterogeneity.^29^

Accessing such detailed structural information from SFG signals, however, can be highly challenging, necessitating an accurate theoretical description of the source signals. In this respect, spectroscopic investigations often make the assumption of in-plane isotropy to apply significant symmetry simplifications to the description of the detected response.^21,22^ This assumption, however, is not generally valid and may only be true when averaged over larger sample areas where the spectroscopic signatures of local in-plane anisotropy vanish due to the spatial integration. At the molecular scale, the in-plane interactions between adjacent molecules often lead to regions (or domains) with preferential orientational and conformational structures. If this thermodynamic preference for the formation of such (quasi-crystalline) packing motifs is significant enough, it can overcome the associated entropic cost. The length-scale of this crystallinity is finely controlled by the significance of this thermodynamic favorability. The validity of assuming in-plane isotropy in SFG measurements then clearly rides on the balance between the length-scale of these orientational correlations and the range over which the obtained signals are averaged. If the latter is significantly larger than any orientational correlation, then the overall response features the same symmetry properties as an in-plane isotropic film. It is hence clear that, while such an assumption may be widely valid for typical SFG spectroscopy, where the signals are integrated over a laser spot size of the order of tens or hundreds of microns, it is often not valid for microscopic imaging of SFG signals. In this context it is important to note that any absence of in-plane isotropy in SFG imaging is not a limitation of the technique. On the contrary, such regions of in-plane anisotropy are precisely the structural features that we wish to extract and characterize. This, however, clearly requires the widely reviewed and commonly used SFG formulations^21−24^ to be extended to include the study of heterogeneous structures with long-range order.

In this contribution, we show how SFG microscopy measurements (resolving the four-dimensional SFG signals as a function of X- and Y- position, spectral frequency, and sample azimuthal rotation angle) can access this additional information on the heterogeneous anisotropic structure in thin molecular films and directly reveal the spatial variation in their 3D molecular orientations. This is demonstrated on a model system consisting of a phase-separated phospholipid monolayer where, as an extension to our recent work highlighting the capabilities of SFG microscopy,^30^ we extract not only the in-plane molecular directions, but also the spatial distributions of out-of-plane tilt angles and the molecular density. We showcase the detailed insight into the molecular packing structure that can be obtained through access to such a combined set of structural information.

To illustrate how noncanceling in-plane contributions can be significant in systems displaying long-range orientational correlations, we perform SFG microscopy on a mixed lipid monolayer (4:1 DPPC:d_82_-POPC) in the SSS polarization combination using our newly developed SFG microscope, details of which can be found elsewhere.^30,31^Figure 1 shows the resulting hyperspectral image (shown as the primary singular value decomposition, SVD, component) taken within the C–H stretching region (only probing the DPPC lipid). The SSS polarization combination only probes a single tensor element, namely the entirely in-plane YYY component. For systems displaying in-plane isotropy, this tensor element, and thus the entire SSS response, should vanish, which is why SSS is often labeled as a symmetry-forbidden polarization combination.^22,32^ As seen in Figure 1a, however, the SSS image of this domain-separated lipid monolayer shows substantial signal amplitudes across the image, clearly demonstrating that the lipid structure adopts a highly ordered packing arrangement, with long-range in-plane ordering. Specifically, this image shows that there is little cancellation within each pixel, and similar structure in adjacent pixels, thus representing clear microscale in-plane orientational correlations.

**Figure 1:**
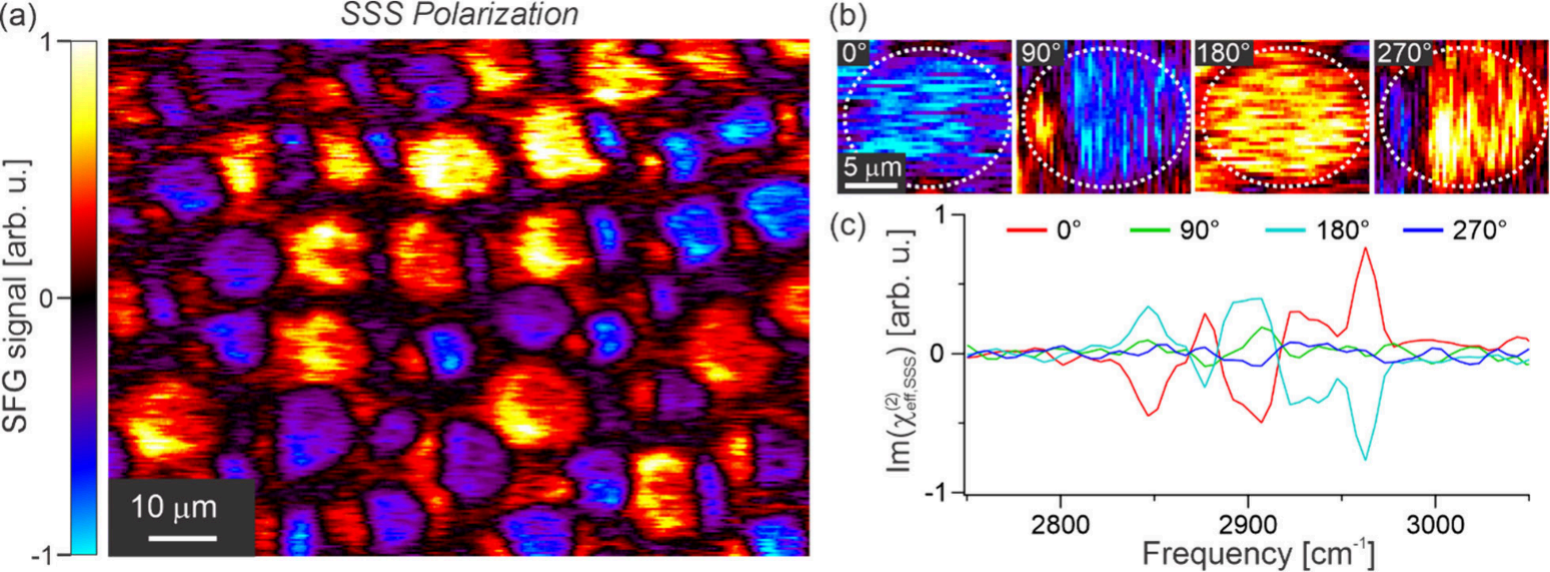
SFG microscopy image of a 4:1 DPPC:d_82_-POPC monolayer in the SSS polarization combination showing (a) a complete wide-field image, (b) images of a single lipid domain for different sample rotations, having been rotated back for comparison, and (c) averaged spectra over the entire image for the four different sample rotations. The SFG images in (a) and (b) represent the singular value decomposition (SVD) maps of the SFG hyperspectral images.

In addition to observing the presence of significant SSS amplitudes from the lipid monolayer, Figure 1a also highlights the different types of heterogeneity in the sample. First, the SSS signals are present in both positive and negative amplitudes. This shows that the in-plane molecular structure significantly varies in orientation across the sample. Furthermore, the SSS signals predominantly arise from roughly circular regions of ∼10 μm diameter. This is consistent with the signals originating from the liquid-condensed (LC) phase which forms such round domains that are enriched in the saturated DPPC lipid and display significant molecular ordering.^33−36^ In contrast, the surrounding liquid-expanded (LE) phase displays less in-plane order of DPPC. Finally, within these circular LC domains, the SSS signals also show both positive and negative amplitudes, with many showing a split character. This demonstrates that not only is there lots of variation in in-plane orientation across the sample, but also across each individual LC domain. Such an observation is consistent with previous findings of these domains displaying a spiraling molecular packing arrangement.^30,37−39^ This is further highlighted through azimuthal-scanning measurements which are shown in Figure 1b for a selected LC domain at 90° intervals (having back-rotated the images for comparison). On inspection, it should first become clear that a 180° rotation of the sample leads to negation of the SSS signals, as expected. Beyond this, however, while 0 and 180° only show signals of the same sign for the entire structure, the 90 and 270° sample rotations are split, demonstrating the differing directionality across the domain.

While this demonstration evidences how assuming in-plane isotropy is far from valid for microscopic imaging of these sorts of molecular samples with microscale ordering, the presence of both positive and negative contributions across the image shows that the full spatial averaging of spectroscopic measurements would involve significant cancellation and improve its validity. Nevertheless, it is worth noting that the cancellation of such signals even when integrating over the ∼100 × 100 μm^2^ image can be imperfect, yielding residual signals. This is shown in Figure 1c which gives the image-averaged spectra for each of the four rotations across the whole sample. If the assumption of in-plane isotropy for the spatial averaged spectrum was accurate for the investigated sample, the spectra would have to be zero for all rotations. From the presented data, however, it becomes evident that this is not the case for 0° and 180°, with both rotations clearly displaying significant residual spectral features including their negation upon 180° rotation, as expected. This highlights that residual in-plane contributions can even enter into the spatially averaged spectrum and should generally not be neglected when investigating systems that can display long-range orientational correlations.

While accessing the in-plane information through SSS measurements is somewhat straightforward given that only a single tensor component contributes to the overall response, elucidating the desired 3D structure of a molecular film requires both the in-plane and out-of-plane components. This hence calls for P-polarized light to be used as it can access such information simultaneously by probing both the x- and *z*-directions in the lab frame. In this sense, the commonly used PPP polarization combination represents a clear choice for SFG measurements. In PPP, however, probing both x- and *z*-directions means a total of 8 components of the second-order susceptibility tensor contribute to the overall response. A full expression for the overall susceptibility accounting for the nonlinear Fresnel factors, *L*, and taking a collinear beam geometry with incidence angle θ′ is given in eq 1.
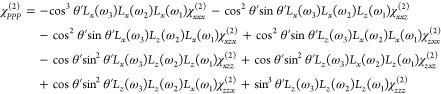
1

This expression can be simplified by noting that off-resonant interactions associated with the two visible beams in the three-wave mixing process have a symmetric polarizability in the absence of any dispersion. This means that the XZX and ZXX tensor components essentially become equal, as do the XZZ and ZXZ (with the order of indices here following the convention of SFG, visible upconversion (UC), IR). Therefore, given a collinear beam geometry and opposite signs of these contributions in eq 1, these four terms will cancel, leaving just four unique tensor components, as in eq 2.
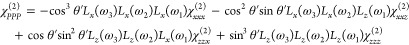
2

Although the analysis of the overall PPP response is hampered by the potentially non-negligible contributions from all four surviving tensor components in eq 2, describing each susceptibility contribution in terms of molecular hyperpolarizabilities through an Euler transformation (converting between the molecular and lab frames) allows the overall response to be written simply in terms of the set of molecular coordinates. While there are many ways to define such a coordinate transformation, we will use the following description that is shown in Figure 2a. In this definition, a rotation angle of ϕ = 0 places the molecular *c*-axis in the *x*–*z* plane, thus aligning with the probing P-polarized E-field direction.

**Figure 2:**
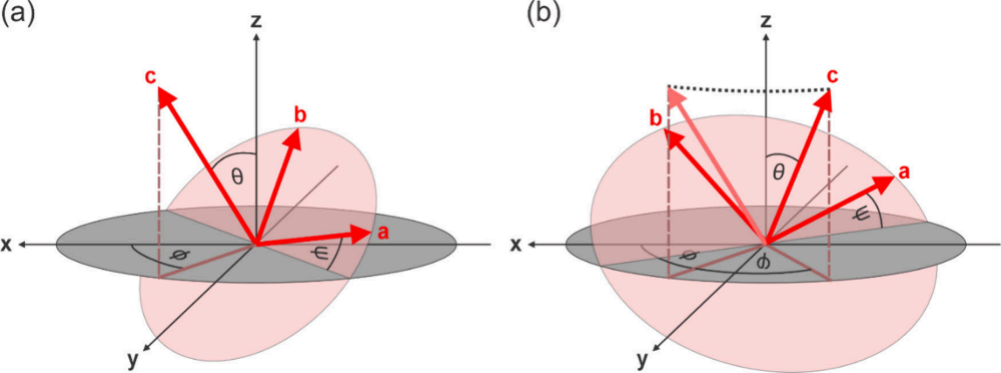
Schematic Euler transforms between the molecular frame (a, b, c) and the lab frame (x, y, z) showing (a) the basic transformation between these two frames in terms of three Euler angles, (θ, ϕ, ψ), and (b) the same transformation including a sample azimuthal rotation angle, φ.

Despite this simplification, the overall PPP response nevertheless still contains both in-plane and out-of-plane contributions which cannot be separated from an individual measurement. Furthermore, without their separation, the two unknown parameters which define the 3D orientation of the molecule, namely the in-plane direction angle, ϕ, and the out-of-plane tilt angle, θ (with the twist angle being considered as a conformational distortion), cannot be disentangled. The solution to this, however, is to perform azimuthal-scanning measurements, thus recording a 4D SFG data set (pixel position in X and Y, vibrational frequency, and azimuthal angle). Figure 2b thus extends the Euler transformation by adding a sample azimuthal rotation angle, φ, with the matrix describing this overall coordinate transformation being given in eq 3.
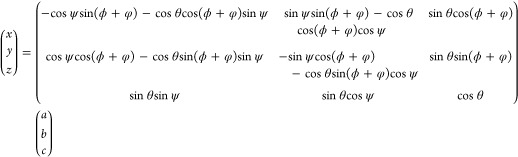
3

For the commonly studied C–H stretches that feature in a wide range of thin films including the lipid systems that have been discussed above, the main resonances to consider are the CH_3_ and CH_2_ symmetric and antisymmetric stretches. Due to their prominence in SFG spectra and lack of cancellation within each alkyl chain (given that there is only a single contribution from each chain), we will use the CH_3_ groups to show here how the structural properties of the film can be unraveled using the rotational analysis.

For the CH_3_ group, it is common to assume local C_3v_ symmetry which essentially neglects any vibrational coupling to the rest of the alkyl chain.^15,21,40^ While deviations from this assumption have been previously noted,^41,42^ with C_S_ being a more accurate symmetry group, the descent in symmetry from C_3v_ to C_S_ in such cases only represents a small perturbation, meaning the different resonances can often be effectively treated within the C_3v_ framework without significant consequences. This simplifies the overall expressions by reducing the total number of nonzero hyperpolarizability components from 14 to 11 and yields further equalities between them.^43^ For further simplicity, we will only consider the symmetric stretch (*r*^*+*^) as it is independent of the conformational twist angle (for which little is known about the orientational distribution),^41^ sufficiently isolated in frequency so as to warrant easy characterization, and yields sufficient information to completely determine the molecular orientations within the film. Within the C_3v_ description (neglecting the acc and cac contributions and equating aac with bbc), the four surviving tensor components contributing to the PPP response can be written as in eqs 4–7, substituting in the hyperpolarizability ratio, , with ρ being the molecular density.
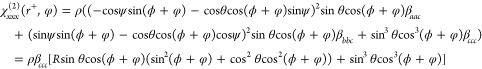
4
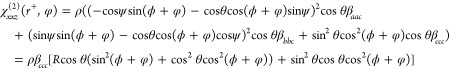
5
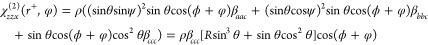
6
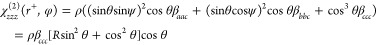
7

Note that the susceptibilities are expressed as a function of sample rotation φ. As a consequence, if the SFG data is recorded for a set of azimuthal rotation angles, φ, the result can be expressed in terms of azimuthal frequencies, *f*, by applying Fourier transformations to each tensor component, yielding eqs 8–11.
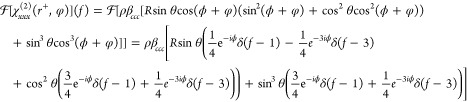
8
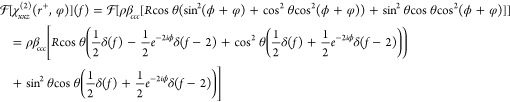
9
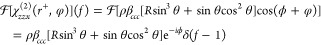
10
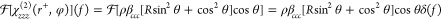
11

For the methyl group of aliphatic alkyl chains, the value of the hyperpolarizability ratio is well-known from polarized Raman measurements, typically with a value of R = 2.22 (based on a value of the hyperpolarizability derivative ratio, *r* = 0.14).^21^ Therefore, the three unknown structural properties, the two Euler angles, θ and ϕ, as well as the molecular density, ρ, can be perfectly extracted from considering only the 0-fold and 1-fold azimuthal frequency responses which are given by eqs 12 and 13.
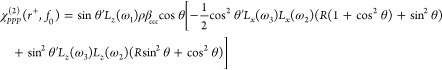
12
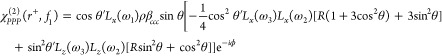
13

As shown in eqs 12 and 13, the 0-fold response is entirely real whereas the 1-fold response is generally complex. Therefore, their combination provides the three observables required to separate the molecular density and two Euler angles. In fact, the in-plane Euler angle, ϕ, is trivially extracted just from the phase of the 1-fold response. Thereafter, combining the magnitudes of the 0- and 1-fold responses (which are independent of ϕ) can separate the tilt angle, θ, from the molecular density (e.g., their ratio is purely a function of θ). It is important to note that, while the structural information can be perfectly extracted with only the 0-fold and 1-fold responses, this necessitates that they are isolated from the higher order frequency contributions. eqs 8–11 clearly show that the overall response contains up to and including a 3-fold azimuthal frequency component, and thus that at least a 60° angular resolution is required for such extraction based on the Nyquist theorem.

By applying the analytical descriptions given above to the SFG microscopy images of the phase-separated lipid monolayers shown previously,^30^ the molecular structure of the film can be characterized pixel by pixel beyond just the in-plane direction that was the focus of the previous investigation. This was achieved by incorporating eqs 12 and 13 into an in-house numerical solver based on the Levenberg–Marquardt least-squares algorithm which inputs the three images (real part of the 0-fold and both real and imaginary parts of the 1-fold response for the methyl symmetric stretch) and outputs the in-plane direction, tilt angle, and molecular density. Figure 3a shows images of these three extracted values for the LC lipid domains. On first inspection of the map of in-plane rotation angles (Figure 3a, right-hand image), the LC domains clearly present long-range in-plane ordering with a curved molecular packing structure, as expected.^30^

**Figure 3:**
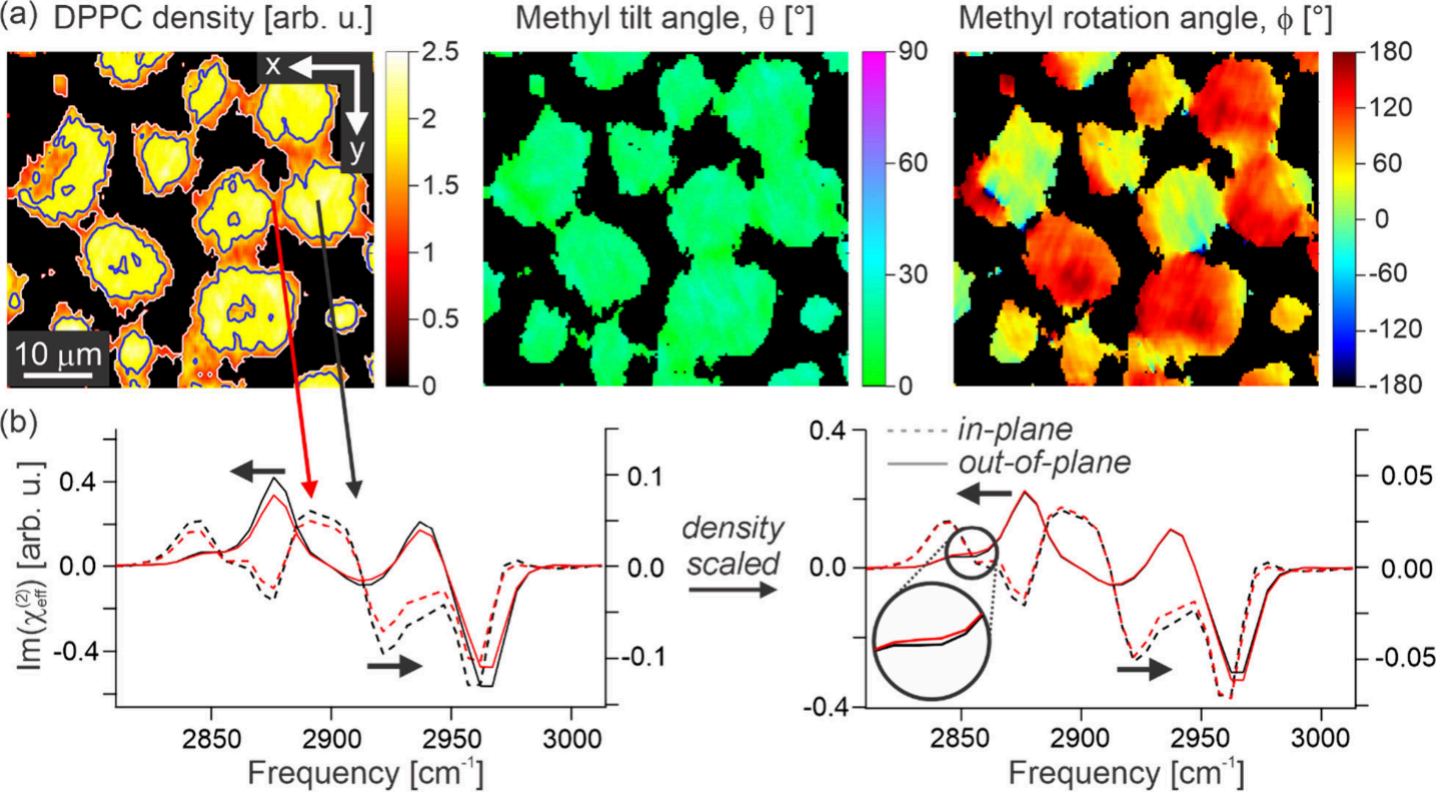
Structural analysis of a 4:1 DPPC:d_82_-POPC monolayer. (a) Images of the molecular density of DPPC, methyl tilt angle, and its in-plane direction angle for the LC lipid domains. (b) Out-of-plane (solid lines, left axis) and in-plane (dashed lines, right axis) spectral components of the overall PPP response, taken as the 0-fold and 1-fold contributions, respectively, for both high (black) and low (red) DPPC density regions in the LC domains, as indicated by the contours in the density map in (a). Also shown are the same spectra calculated by removing the impact of the density variations (again with the out-of-plane spectra being shown on the left axis, and in-plane on the right axis).

Beyond this in-plane directionality, the maps in Figure 3a for the density and methyl tilt angle give a more detailed insight into the specific structure and heterogeneity of the lipid film. Specifically, the density map reveals substantial heterogeneity within the LC domains, particularly highlighting a reduction in DPPC density toward the boundary, i.e., on transitioning toward the LE phase. In contrast, the map of methyl tilt angles is largely homogeneous and shows no clear correlations to the density or in-plane rotation angle. This reveals that the variations in both density and in-plane direction surprisingly have little-to-no impact on the chain tilt structure.

For further insight into the correlation between the apparent DPPC density and packing structure, the in-plane (IP) and out-of-plane (OP) spectral components of the overall PPP response can be compared, being defined as the spectral contributions to the 1- and 0-fold azimuthal frequencies, respectively. The spatially averaged IP (dashed lines) and OP (solid lines) spectra are shown in Figure 3b for regions with relatively high (black) and low (red) density, as indicated by the contour lines in Figure 3a. On initial inspection, both the IP and OP spectra are lower in amplitude for the lower density regions, as expected, but overall have very similar line-shapes, indicating that any structural changes must be minor. For a better comparison, Figure 3b also shows the IP and OP spectra for the two regions having accounted for the density variations. On close inspection of the density-scaled OP spectra, the low-density region has a very slightly larger CH_2_ contribution at ∼2850 cm^–1^ (symmetric stretch^22^), although the difference is very small. However, compared to the exceptional overlap at other frequencies, this difference appears significant. Such an increase in CH_2_ signals may be indicative of a slightly higher density in conformational (gauche) defects that reflects the lower density. Additionally, and more significantly, the ratio between the symmetric and antisymmetric CH_3_ amplitudes is also reduced for the low-density region, which is indicative of a change in either the tilt angle and/or twist angle. From inspecting the tilt angle map, however, there is no indication of an increase in tilt angle for the low-density regions, suggesting that the slight change in methyl symmetric-to-antisymmetric ratio is most likely associated with the molecular twist angle distribution. This difference hence suggests a change to the intermolecular landscape that alters the thermodynamic preference for specific methyl conformations (twists), and thus is likely associated with a change in molecular order. This qualitative analysis is further supported by a quantitative comparison of the fitted spectra given in the Supporting Information.

While the OP spectra in Figure 3b show very minimal disparities between the low- and high-density regions, the IP spectra have somewhat more noticeable differences. Specifically, despite the scaling, the low-density region has generally lower amplitude (see Supporting Information), suggesting an overall smaller amount of IP order, thus aligning with the apparent change in conformational twist angle discussed above. This further aligns with expectations as both the LC and LE phases were shown previously to yield clear OP SFG signals, indicating significant OP ordering in both phases.^30^ On the other hand, the LE phase has a more disordered packing structure with lesser IP order, as is clearly shown in the SSS amplitudes in Figure 1a. Therefore, while the transition in OP order between the two phases may only be minor, the change in IP order must be more significant. What is notable here is that this transition appears not to be particularly sharp at the domain boundary, but spans a microscale region where the DPPC density is also lowered. This further suggests a clear correlation between the local density of the saturated DPPC lipid and the amount of IP packing order in the system, once again aligning with expectations due to the ability for DPPC to more tightly pack and form stronger intermolecular interactions.^33−36^

Finally, with the methyl tilt angle ranging from ∼10–25°, one can also infer the tilt structure of the chain. As shown in Figure 4a, there are two limiting cases connecting the chain tilt angle, α, to the methyl tilt, with case 1 being the chain tilting in the same direction as the methyl group, and case 2 being opposing directions. In these two cases, the methyl tilt angle is simply given by eq 14, where τ is the tetrahedral bond angle, with their relationships being plotted in Figure 4b.
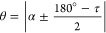
14

**Figure 4:**
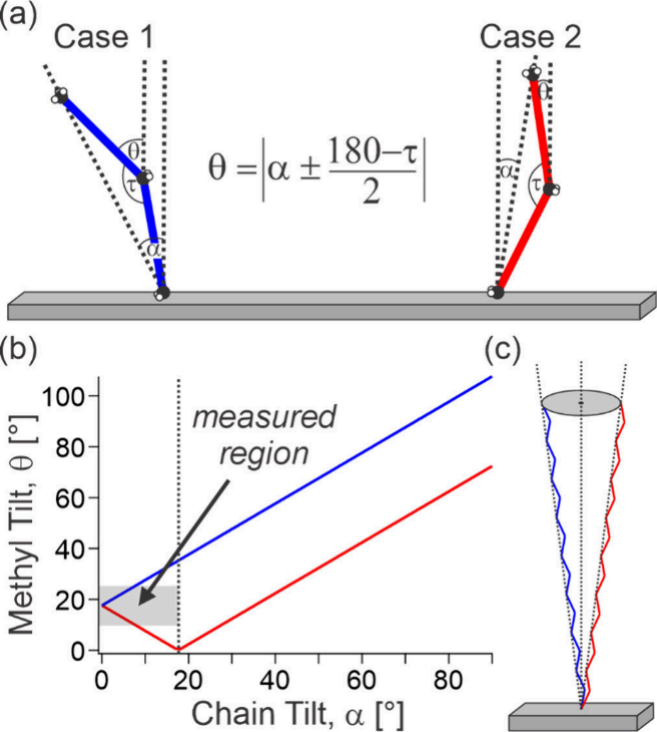
Structural analysis of the chain tilt angles. (a) Limiting cases connecting the methyl tilt angle to the chain tilt angle, being either in the same direction (case 1, blue) or opposite direction (case 2, red). (b) Plot of the relationship between the methyl and chain tilt angles for both cases, also indicating the region of the graph where the chain tilt angle is well-defined (<∼18°) and the region also corresponding to the measured range of methyl tilt angles (∼10–25°). (c) Schematic structure of the cone within which the tails must be contained based on the measured values.

Figure 4b clearly shows that the link between the methyl and chain tilt angles is not generally well-defined, except for  where the function becomes one-to-one (with methyl tilt as the input). However, given that the monolayers are formed with a very high average surface concentration (∼1.5 molecules nm^–2^), the packing within the LC domains should result in almost upright lipid tails. It is thus reasonable to restrict the chain tilt angle to this region where an exact correspondence link to the methyl tilt angle can be reached. With this restriction, the measured range of the methyl tilt angles (∼10–25°) yield a chain tilt angle of <8°. In fact, as the two discussed cases are limiting values, even if the tilt direction is not perfectly within the molecular plane, then the chain tilt will be within this range. It can thus be concluded that the tails must be contained within an 8° cone, as schematically shown in Figure 4c.

In summary, we have discussed the theoretical considerations in SFG measurements that become important when probing heterogeneous molecular structures that can display long-range orientational correlations, differing from the theory conventionally used in SFG spectroscopy studies. Based on this framework, we show that azimuthal-scanning (AS) measurements can be used to disentangle in-plane and out-of-plane contributions and gain access to the full 3D molecular orientation. This higher dimensional imaging methodology is then demonstrated on the mixed lipid monolayer system where the in-plane direction and out-of-plane tilt angle are extracted along with variations in molecular density across the surface. This analysis revealed clear variations in density across the LC lipid domains, particularly on approaching their boundary to the LE phase. Through a spectral analysis of these regions, the subtle structural differences associated with this transition are analyzed, noting that the reduction in molecular density is accompanied by little change in out-of-plane structure, but a defined decrease in in-plane ordering. While such a structural change is expected on transitioning to the more disordered LE phase, here we show that these changes are not sharp, but occur over a microscale region (∼1–2 μm) at the domain edge. Finally, through an analysis of the methyl tilt angles, the chain tilt structure is determined to be contained within a narrow cone of 8° inclination, thus representing an exceptionally upright packing structure. This showcase example of the analysis of a lipid monolayer thus highlights the possibilities of 3D structural characterization using AS-SFG microscopy. This hence opens up possibilities to elucidate the structure of a wide variety of thin molecular films and gain much deeper insight into the connection between heterogeneous molecular structure and macroscopic properties. This includes answering questions surrounding the synergistic interactions between different constituents, the effect of temperature and phase transitions, and the role molecular chirality on the macroscopic behavior.

The potential of this vibrational imaging technique, however, clearly goes beyond the structural insight gained on the highlighted example. The presented data illustrates how sensitive SFG microscopy is to structural motifs such as molecular packing, ordering, configuration, conformation, orientation, etc., making it a unique and powerful probe of local molecular structure in heterogeneous systems. Furthermore, it also allows highly congested spectra to be decomposed into modes with different azimuthal dependencies. One can then even envisage a future direction being further expanding the dimensionality of SFG microscopy and increasing its elucidative potential to address far more complex samples that are relevant e.g. in biology. The AS approach is nothing other than a modulation of the probing polarization which could easily be extended to include more degrees of freedom, such as relative beam polarizations or incidence angles, all of which highly modulate the output signals. Obtaining such a high-contrast, high-dimensional dataspace would be a perfect candidate to employ machine learning algorithms to draw connections between the appearance of particular polarization and azimuthally dependent spectral features in specific spatial locations and macroscopic properties. Such connections could be promising for many applications in the biological and physical sciences, such as disease-screening or the development of novel polymeric materials.

## Experimental Methods

### Sample Preparation

The lipids 1,2-dipalmitoyl-*sn*-glycero-3-phosphocholine (DPPC, > 99%) and d_82_-1-palmitoyl-2-oleoyl-glycero-3-phosphocholine (dPOPC, > 99%) were obtained from Avanti Polar Lipids (Alabaster, AL, USA) and dissolved in chloroform (99.0–99.4%, VWR International GmbH, Darmstadt, Germany) at a concentration of 1 mg mL^–1^.

The mixed lipid monolayers were formed by first premixing the two lipids in a 4:1 mass ratio (DPPC to dPOPC) and subsequently depositing them dropwise onto the surface of a PTFE Langmuir–Blodgett trough (MicroTrough G1, Kibron, Helsinki, Finland) filled with ultrapure water (Milli-Q, 18.2 MΩ cm, < 3 ppb total organic carbon). The films were left for ∼5 min immediately after deposition to allow the chloroform to evaporate, then compressed to 20 mN m^–1^ with a barrier speed of 10 mm min^–1^, and left for a further 2 h to equilibrate and oxidize due to ambient ozone.^17^ Prior to deposition, the trough was cleaned with both chloroform and ultrapure water, and the water surface was repeatedly aspirated until the surface pressure was within 0.1 mN m^–1^ at full compression. Additionally, the platinum pressure sensor was flamed and rinsed with both chloroform and ultrapure water to remove any contamination.

For the microscopy measurements, the monolayers needed to be immobilized to avoid both their natural dynamical behavior as well as that driven through Bénard-Marangoni convection due to local heating of the subphase water.^44^ This was achieved through Langmuir–Blodgett deposition using a LayerX dipper at a rate of 2 mm min^–1^ (Kibron, Helsinki, Finland) onto ultraflat fused silica windows (Korth Kristalle, Altenholz, Germany, 5 mm thick, 25.4 mm diameter, and <2 nm surface roughness) which were cleaned using both chloroform and ultrapure water, as well as exposed to UV-ozone for at least 30 min (UV/Ozone ProCleaner Plus, BioForce Nanosciences, Virginia Beach, VA, USA).

### SFG Microscope and Data Treatment

Full details of the SFG microscope and the data analysis used in this work can be found in our previous publication.^30^ In short, the heterodyned SFG microscope operates fully in the time-domain using a home-built interferometer^45^ and the broadband IR and visible pulses generated through optical parametric amplification of the 800 nm output from a Ti:sapphire laser. The incident pulses (IR, visible, and local oscillator reference) are input collinearly into a custom-made reflective objective after which they are filtered, temporally aligned, and focused onto an electrically cooled CCD camera, also implementing paired-pixel balanced imaging for noise suppression.^31^

The hyperspectral SFG images are individually recorded from −300 to 3000 fs in 2 fs steps and referenced in phase and amplitude by a similar scan run on z-cut quartz from −300 to 300 fs. The presented data represent the coaverage of multiple scans (at least four per azimuthal angle). The resulting images are then further treated by removing the dark counts and a linear (nonresonant) baseline. The resulting hyperspectral SFG images are then back-rotated for each sample rotation angle and subsequently overlapped to generate a 4D image (SFG signal as a function of time delay and sample rotation angle). This is then Fourier-transformed into azimuthal frequencies, and the spectral information obtained by a further Fourier transform of the time-domain axis, ultimately yielding rotational magnitude and phase information as a function of vibrational frequency.

### Spectral Analysis

The analysis of the hyperspectral SFG images was performed in Matlab using an in-house fitting procedure to numerically solve eqs 12 and 13 for the DPPC density, methyl tilt angle, and in-plane direction angle. This procedure utilized the Levenberg–Marquardt least-squares algorithm based on input matrices of the real part of the 0-fold azimuthal response as well as both real and imaginary parts of the 1-fold response. For the presentation of the SSS data, an SVD algorithm was used, as described previously,^30^ to reduce the hyperspectral image into its principle components, where only a single component was found to adequately represent the data and substantially reduce the noise contributions.

For the comparison of the in-plane (IP) and out-of-plane (OP) spectral components of the overall PPP response, the 1-fold and 0-fold azimuthal frequencies of the 4D SFG images as functions of spectral frequency are extracted. These are then spatially averaged based on a predefined mask (in this case generated from the density image shown in Figure 3a), also considering the orientation dependence of the IP contribution that is defined through the 1-fold phase.

## Supporting Information Available

Quantitative spectral fitting and analysis (PDF)
Transparent Peer Review report available (PDF)
