# Supplementary material for: Extracting the Heterogeneous 3D Structure of Molecular Films Using Higher Dimensional SFG Microscopy
Source: J Phys Chem Lett. 2024 Oct 22;15(43):10849–57. doi: 10.1021/acs.jpclett.4c02679 (PMC11533227; doi:10.1021/acs.jpclett.4c02679)
Supplement: Supplementary file 1 — jz4c02679_si_001.pdf [file jz4c02679_si_001.pdf]

Supporting Information

Extracting the Heterogeneous 3D Structure of Molecular Films using Higher Dimensional SFG Microscopy

Alexander P. Fellows, Ben John, Martin Wolf, and Martin Thämer*

Fritz-Haber-Institut der Max-Planck-Gesellschaft, Faradayweg 4-6, Berlin, 14195, Germany

*Corresponding Author

thaemer@fhi-berlin.mpg.de

Spectral Fitting

The out-of-plane (OP) and in-plane (IP) spectra (scaled for density) shown in Figure 3 in the main text were fitted with Lorentzian functions based on Eq. S1,

$$\chi_n^{(2)} = \frac{A_n}{\omega_n - \omega - i\Gamma_\mathrm{n}} \qquad (S1)$$

where A_n is the amplitude, ω_n the resonant frequency, and Γ_n the damping coefficient.[1,2] This was achieved using an in-house numerical solver based on the Levenberg-Marquardt least-squares algorithm, where the four spectra (IP and OP for both the high-, H, and low-, L, density regions) were simultaneously fitted using identical center frequencies and damping coefficients, only allowing their amplitudes to vary. The fitted spectra are presented in Figure S1, with the corresponding fitting parameters given in Table S1.

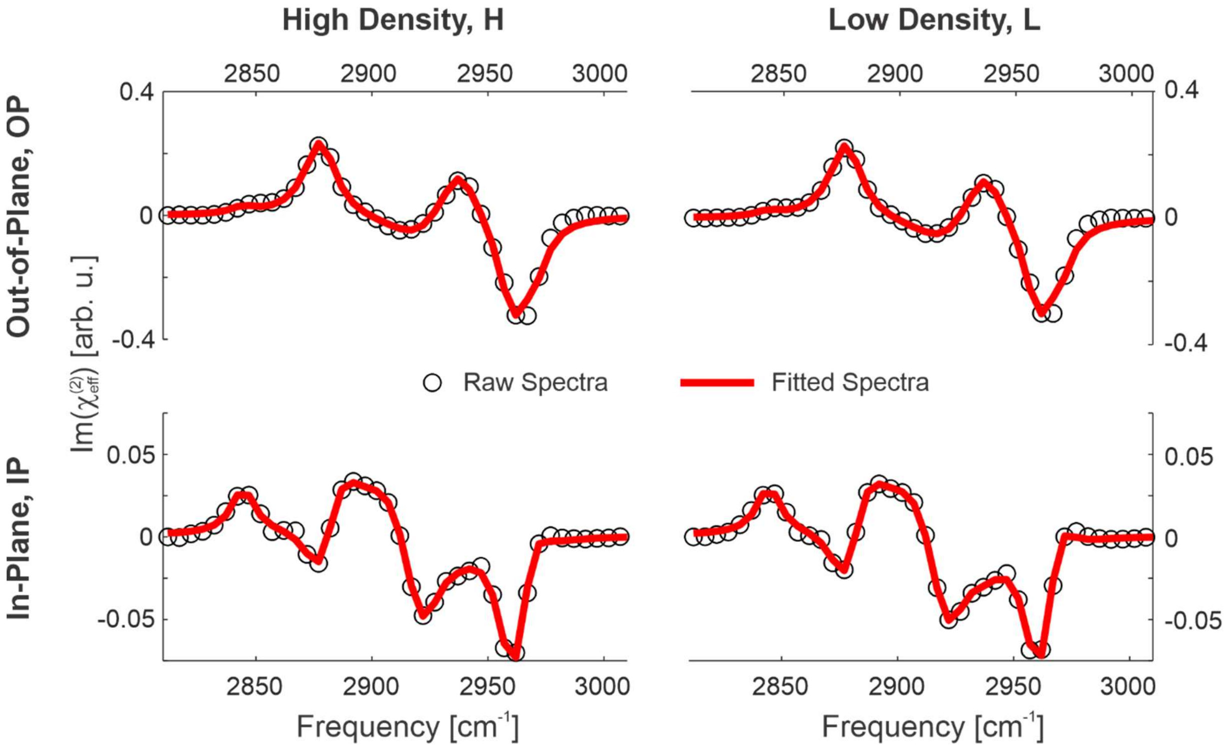

Figure S1: Out-of-plane (OP) and in-plane (IP) spectra for both the high (H) and low (L) density regions, as shown in Figure 3 in the main text, along with their corresponding fitted spectra based on the Lorentzians given by the parameters in Table S1.

Table S1: Fit parameters for the individual Lorentzian bands used for the fitted spectra shown in Figure S1. The values are given for each band along with their assignment based on previous values from the literature.[1,3] *OP: out-of-plane, IP: in-plane, H: high density region, L: low density region, FR: Fermi resonance.*

Band	Assignment	Frequency, ω_i	Damping Coefficient, Γ_i	Amplitude, A_i			
				OP		IP	
				H	L	H	L
1	d^+	2844	6	0.11	0.14	0.18	0.17
2	r^+	2878	9	2.15	2.20	-0.46	-0.40
3	d^+_{FR}	2886	11	-0.13	-0.13	0.40	0.39
4	-	2915	20	-0.84	-0.87	1.75	1.73
5	-	2921	12	-0.60	-0.56	-1.51	-1.50
6	r^+_{FR}	2938	10	1.87	1.92	-0.20	-0.11
7	r^-_{IP}	2961	8	-2.15	-2.26	-0.67	-0.66
8	r^-_{OP}	2970	7	-0.80	-0.90	0.19	0.15

As mentioned in the main text, analysis of the spectra shows distinct differences. For the OP spectra, these differences are primarily associated with the ratio of the CH_3 symmetric (r^+) and CH_3 antisymmetric (r^-) stretches, but also with the relative amplitude of the CH_2 symmetric stretch. Specifically, the low-density region was found to have a larger CH_2 signal as well as a smaller symmetric-to-antisymmetric CH_3 ratio. This qualitative analysis is supported by the fitted amplitudes, which show a ~30% increase in the CH_2 symmetric stretch along with a ~5% reduction in the symmetric-to-antisymmetric CH_3 ratio. For the IP spectra, comparison of the raw spectra in the main text showed a defined decrease in amplitude for the low-density region (cf. high-density). Once again, this is supported by the quantitative fitting. All bands are fitted with lower amplitudes (in magnitude) for the low-density region and, while for some the difference is only marginal, others have a more pronounced decrease, particularly the main CH_3 bands (r^+, r^+_{FR}, and both r^- bands), which is indicative of lower in-plane order within this region.

References

(1) Lambert, A. G.; Davies, P. B.; Neivandt, D. J. Implementing the Theory of Sum Frequency Generation Vibrational Spectroscopy: A Tutorial Review. *Appl. Spectrosc. Rev.* **2005**, *40* (2), 103–145. https://doi.org/10.1081/ASR-200038326.

(2) Shen, Y. R. *Fundamentals of Sum-Frequency Spectroscopy*; Cambridge Molecular Science; Cambridge University Press: Cambridge, 2016.

(3) Fellows, A. P.; John, B.; Wolf, M.; Thämer, M. Spiral Packing and Chiral Selectivity in Model Membranes Probed by Phase-Resolved Sum-Frequency Generation Microscopy. *Nat. Commun.* **2024**, *15* (1), 3161. https://doi.org/10.1038/s41467-024-47573-1.
