# Supplementary material for: Extracting the Heterogeneous 3D Structure of Molecular Films Using Higher Dimensional SFG Microscopy
Source: J Phys Chem Lett. 2024 Oct 22;15(43):10849–57. doi: 10.1021/acs.jpclett.4c02679 (PMC11533227; doi:10.1021/acs.jpclett.4c02679)
Supplement: Supplementary file 2 — jz4c02679_si_002.pdf [file jz4c02679_si_002.pdf]

jz-2024-02679q.R1

Name: Peer Review Information for "Extracting the Heterogeneous 3D Structure of Molecular Films using Higher Dimensional SFG Microscopy"

First Round of Reviewer Comments

Reviewer: 1

Comments to the Author

In the submitted manuscript, the authors utilized phase-sensitive sum-frequency generation spectroscopy technique to reveal the 3D orientation of molecular films, whose structural information is very important for improving and designing advanced molecular films. The sample rotation dependent SFG spectra revealed the 3D conformation of C-H bonds. The experiments are well designed and the interpretations of results are carefully considered. Therefore, I believe that this manuscript provides novel knowledge regarding the application of SFG spectroscopy on molecular films and is publishable from JPC Lett as it is.

Reviewer: 2

Comments to the Author

The authors proposed to solve in-plane anisotropy problems based on the newly-developed SFG microscopy signals. This reviewer likes the idea very much. However, this manuscript did not give any physical insights into molecular films. Instead, they focused on extending the development of SFG microscopy. This manuscript is publishable in J. Phys. Chem A with considering as follows:

1. The traditional Euler transformation defines azimuthal angle. However, the authors defined additional sample azimuthal rotation angle without any physical meaning, leading a confusing expression in Eq. 3. This reviewer strongly suggested that the addition azimuthal angle not be presented.

2. The follow-up of question 1. As long as the angle (phi) was correctly defined, one should not need the addition azimuthal angle. What are the physical meanings for 0, 90, 180, 270 of a new phi, which should be clarified in an updated version?

3. This reviewer suggested that the authors stress the importance for doing transformation from azimuthal angle to azimuthal frequencies, f. I guess that it is a mathematical step.

4. In eqns. 12 and 13, they look straightforward due to 0 st and 2 nd in the azimuthal frequencies. However, how many data does one need to do in azimuthal angle domain? It turns out that they are independent of azimuthal angles. But it must be a better way to express the transformation in Eqs 8-11.

5. Now the definition of angle of phi is very important.

6. The physical meanings of Figure 3 (a).

Reviewer: 3

Comments to the Author

The authors present a new method for analyzing SFG images for extracting information about interfacial morphology, molecular orientation, and molecular organization with spatial resolution. Their method incorporates azimuthal scanning into the conventional description of the second order susceptibility response. The azimuthal rotation introduces a frequency dependence in the susceptibility, allowing for extraction of molecular density and Euler angels, by Fourier transform of the susceptibility tensor elements. The authors use this method to probe a lipid monolayer made of 4:1 DPPC:d82-POPC. The authors show that the monolayer forms discrete condensed and expanded domains. They further extract the population density, methyl tilt angle, and methyl rotation angle of the terminal methyl group of DPPC. They find an average methyl tilt angle of ~10-25° and chain tilt angle cone of < 8°.

In this work, the authors demonstrate their new method can resolve structural differences in heterogeneous interfaces using SFG imaging. The manuscript should be accepted for publication after the authors addressing the following concerns:

1. Equation 12 may have an error. The equation has a negative sign at the front of the expression. The authors may want to check whether this negative sign should be associated with the first term inside the square brackets (as in Equation 13). This potential error may have significant impacts on the conclusion about molecular orientations and structures.

2. The authors should consider moving the equation number labels to align with the far-right margin for clarity.

3. The authors discuss relatively small variations in lineshape shown in Figure 3b (right, black circled) for the in-plane and out-of-plane spectra. Because the comparison involves scaling the spectral intensities and because the difference is very subtle, the authors may need to provide more quantitative description about their comparison to support the analyses. The authors should consider fitting the spectra and comparing the fitting parameters in a quantitative manner.

4. In Figure 3b: It is not clear what the right-hand axes are.

5. The authors should clarify what they mean for "in-plane" and "out-of-plane" in Figure 3b: whether they refer to the in-plane and out-of-plane components of the ppp-polarization SFG response or whether they refer "in-plane" as the sss polarization and "out-of-plane" as the ppp polarization. Although the former is more likely, the author may want to clarify.

6. The authors only cite ref. 30 (Nat. Comms. 2024) without providing much description about how they obtain the in-plane and out-of-plane signals from the 4D SFG data stack. The authors should consider providing a brief overview about how they extract the two signals from the data. This overview should help readers to better understand what steps and principles are involved in applying their data analysis method.

Author's Response to Peer Review Comments:

Reviewer: 1

Recommendation: This paper represents a significant new contribution and should be published as is.

Comments:

In the submitted manuscript, the authors utilized phase-sensitive sum-frequency generation spectroscopy technique to reveal the 3D orientation of molecular films, whose structural information is very important for improving and designing advanced molecular films. The sample rotation dependent SFG spectra revealed the 3D conformation of C-H bonds. The experiments are well designed and the interpretations of results are carefully considered. Therefore, I believe that this manuscript provides novel knowledge regarding the application of SFG spectroscopy on molecular films and is publishable from JPC Lett as it is.

We would like to sincerely thank the reviewer for taking the time to provide us with their expert review of our manuscript. We very much appreciate the positive comments towards our efforts.

Reviewer: 2

Recommendation: Reconsider as an article in The Journal of Physical Chemistry A/B/C.

Comments:

The authors proposed to solve in-plane anisotropy problems based on the newly-developed SFG microscopy signals. This reviewer likes the idea very much. However, this manuscript did not give any physical insights into molecular films. Instead, they focused on extending the development of SFG microscopy. This manuscript is publishable in J. Phys. Chem A with considering as follows:

We thank the reviewer for their valuable time in reviewing our manuscript and providing us with their feedback. While we are glad that the reviewer appreciated the idea behind this paper, we strongly disagree with their assessment that the manuscript solely focused on the development of SFG microscopy without giving any physical insights into molecular films. We acknowledge that a significant part of the manuscript is centered around how SFG microscopy can be used through azimuthal-dependent measurements to directly probe the heterogeneity in anisotropic molecular structure. After this theoretical discussion, however, the described principles are directly applied to an interesting and highly relevant sample system, namely phase-separated lipid monolayers, to extract previously unexplored structural aspects about their formed domain structure. In fact, the entire second half of the paper, including Figures 3 and 4, solely focuses on the structural insight of this system, with novel findings about the structure of the domain edges and the connection between the molecular density and orientational parameters (Euler angles).

1. *The traditional Euler transformation defines azimuthal angle. However, the authors defined additional sample azimuthal rotation angle without any physical meaning, leading a confusing expression in Eq. 3. This reviewer strongly suggested that the addition azimuthal angle not be presented.*

The reviewer seems to have not understood the necessity for the added sample rotation angle. While they are correct that the Euler transformation defines an azimuthal angle, this is for the transformation from the local molecular coordinates to the sample-frame coordinates, with the set of (likely different) azimuthal Euler angles for all of the molecules on the sample describing the macroscopic molecular structure. If then the sample is rotated within the lab-frame (defined by the input laser direction), then each of these individual azimuthal Euler angles for each molecule are modulated by the same amount, i.e. the sample rotation angle. It is this rotation angle that is exploited to obtain the specific molecular Euler angles relative to the sample frame coordinates. Without defining the sample rotation angle, the rotational Fourier transform simply cannot be performed.

2. *The follow-up of question 1. As long as the angle (phi) was correctly defined, one should not need the addition azimuthal angle. What are the physical meanings for 0, 90, 180, 270 of a new phi, which should be clarified in an updated version?*

As we mention above, this is simply the sample rotation angle which is used to modulate the individual SFG responses from the molecules in the lab-frame. As this issue was not raised by either of the other two reviewers, we believe that the definition of this angle is sufficiently clear in the manuscript. In fact, the method is first introduced as an “azimuthal-scanning” approach exploiting

the "sample azimuthal rotation angle". Thereafter, the rotationally dependent SSS measurements are clearly described as being "for different sample rotations" and, when introducing the new azimuthal angle φ in the extended Euler transformation to connect the molecular frame not to the sample frame, but rather to the lab frame, we explicitly state once again that this is the "sample azimuthal rotation angle". We hope that this, along with the above description of the importance of this angle, has suitably clarified this point for the reviewer.

3. *This reviewer suggested that the authors stress the importance for doing transformation from azimuthal angle to azimuthal frequencies, f. I guess that it is a mathematical step.*

We are somewhat confused by this comment as the conversion from azimuthal angles to azimuthal frequencies is nothing other than a Fourier transformation (as clearly stated in the manuscript) i.e., a mathematical step that is incredibly widespread in the physical sciences. For better clarity, we have added a section on page 15 in the experimental methods briefly describing the data treatment procedure in generating the 4D SFG images which are a function of spectral frequency and azimuthal frequency.

4. *In eqns. 12 and 13, they look straightforward due to 0 st and 2 nd in the azimuthal frequencies. However, how many data does one need to do in azimuthal angle domain? It turns out that they are independent of azimuthal angles. But it must be a better way to express the transformation in Eqs 8-11.*

We thank the reviewer for raising this question as it is indeed an important point about the number of azimuthal angles required for such analysis as this directly connects to the Nyquist theorem. We have made changes to the manuscript on page 10 to emphasize this point. Regarding their comment about the independence on azimuthal angles in the transformations in Eqs. 8-11, we again would like to thank the reviewer for making this point as it led us to the typo where we state on page 10 that the magnitudes of the 0- and 1-fold responses are independent on , i.e. the sample rotation angle. This was an error, and we of course meant that they are independent on the molecular azimuthal Euler angle, ϕ. This typo has now been corrected.

For the representation of the transforms, we have used the generally accepted method for writing the Fourier transform, represented by $\mathcal{F}$, which takes in the function dependent on azimuthal angles and generates a function dependent on azimuthal frequencies.

5. *Now the definition of angle of phi is very important.*

We are confused by what the reviewer is saying with this comment. As mentioned above, the definition of phi is indeed important, but is nevertheless rigorously and repeatedly defined in the manuscript for optimum clarity.

6. *The physical meanings of Figure 3 (a).*

Again, we are not sure exactly what the reviewer means by this comment. The three images are well-defined, with the first clearly presented as the density of the lipid DPPC, the second being the tilt angle of the methyl group, described by the Euler angle θ, and the third being the azimuthal rotation angle of the methyl group, described by the Euler angle ϕ. This is not only described in the figure, but also in the main text.

Reviewer: 3

Recommendation: This paper is publishable subject to minor revisions noted. Further review is not needed.

Comments:

The authors present a new method for analyzing SFG images for extracting information about interfacial morphology, molecular orientation, and molecular organization with spatial resolution. Their method incorporates azimuthal scanning into the conventional description of the second order susceptibility response. The azimuthal rotation introduces a frequency dependence in the susceptibility, allowing for extraction of molecular density and Euler angels, by Fourier transform of the susceptibility tensor elements. The authors use this method to probe a lipid monolayer made of 4:1 DPPC:d82-POPC. The authors show that the monolayer forms discrete condensed and expanded domains. They further extract the population density, methyl tilt angle, and methyl rotation angle of the terminal methyl group of DPPC. They find an average methyl tilt angle of ~10-25° and chain tilt angle cone of < 8°.

In this work, the authors demonstrate their new method can resolve structural differences in heterogeneous interfaces using SFG imaging. The manuscript should be accepted for publication after the authors addressing the following concerns:

Firstly, we would like to express our gratitude to the reviewer for reviewing our manuscript and providing us with their comments and feedback for its improvement. For each of the points raised, we have replied below and, where appropriate, made changes to the manuscript.

1. *Equation 12 may have an error. The equation has a negative sign at the front of the expression. The authors may want to check whether this negative sign should be associated with the first term inside the square brackets (as in Equation 13). This potential error may have significant impacts on the conclusion about molecular orientations and structures.*

We cannot thank the reviewer enough for drawing our attention to this mistake. They are absolutely correct that the negative sign should only be associated with the first term in the square brackets. This mistake has now been corrected. We can confirm, however, that this was purely a typo in the manuscript and thus has no impact on the presented results.

2. *The authors should consider moving the equation number labels to align with the far-right margin for clarity.*

We thank the reviewer for noting this. It seems to be an issue with the PDF converter used on journal submission as the labels are right-aligned in the submitted .docx file. We will nevertheless ensure that they are clear in the journal proof.

3. *The authors discuss relatively small variations in lineshape shown in Figure 3b (right, black circled) for the in-plane and out-of-plane spectra. Because the comparison involves scaling the spectral intensities and because the difference is very subtle, the authors may need to provide more quantitative description about their comparison to support the analyses. The authors should consider fitting the spectra and comparing the fitting parameters in a quantitative manner.*

We agree that a quantitative analysis of the spectra to support the qualitative assessment was missing from the manuscript. We have thus added the quantitative fitting of the spectra in the

Supporting Information along with a discussion of the fitted amplitudes, showing that they precisely support the qualitative comparisons presented in the main text.

4. *In Figure 3b: It is not clear what the right-hand axes are.*

We thank the reviewer for drawing our attention to this lack of clarity. The two sets of axes are associated with the in-plane (right) and out-of-plane (left) spectra and are indicated by the black arrows in the plots. As this was not sufficiently clear, we have enlarged the arrows in the figure and added this information to the figure caption.

5. *The authors should clarify what they mean for "in-plane" and "out-of-plane" in Figure 3b: whether they refer to the in-plane and out-of-plane components of the ppp-polarization SFG response or whether they refer "in-plane" as the sss polarization and "out-of-plane" as the ppp polarization. Although the former is more likely, the author may want to clarify.*

We thank the reviewer once again for drawing our attention to this missing clarity. They are correct that here we are referring to the in-plane and out-of-plane components of the PPP response. We have clarified this in both the main text on page 11 and figure caption.

6. *The authors only cite ref. 30 (Nat. Comms. 2024) without providing much description about how they obtain the in-plane and out-of-plane signals from the 4D SFG data stack. The authors should consider providing a brief overview about how they extract the two signals from the data. This overview should help readers to better understand what steps and principles are involved in applying their data analysis method.*

We thank the reviewer for this suggestion. We have added two brief sections to the experimental methods on page 15, the first better describing the initial data treatment in generating the 4D SFG matrix as a function of vibrational frequency and azimuthal frequency. The second added section then briefly describes the extraction of the OP and IP spectra from the 0-fold and 1-fold azimuthal components.
